# Consumer-Generated Discourse on Cannabis as a Medicine: Scoping Review of Techniques

**DOI:** 10.2196/35974

**Published:** 2022-11-16

**Authors:** Sedigheh Khademi Habibabadi, Christine Hallinan, Yvonne Bonomo, Mike Conway

**Affiliations:** 1 Department of General Practice Melbourne Medical School Faculty of Medicine, Dentistry and Health Sciences, The University of Melbourne Melbourne Australia; 2 Health & Biomedical Research Information Technology Unit The University of Melbourne Melbourne Australia; 3 School of Computing & Information Systems The University of Melbourne Melbourne Australia

**Keywords:** social media, data mining, internet and the web technology, consumer-generated data, medicinal cannabis, medical marijuana

## Abstract

**Background:**

Medicinal cannabis is increasingly being used for a variety of physical and mental health conditions. Social media and web-based health platforms provide valuable, real-time, and cost-effective surveillance resources for gleaning insights regarding individuals who use cannabis for medicinal purposes. This is particularly important considering that the evidence for the optimal use of medicinal cannabis is still emerging. Despite the web-based marketing of medicinal cannabis to consumers, currently, there is no robust regulatory framework to measure clinical health benefits or individual experiences of adverse events. In a previous study, we conducted a systematic scoping review of studies that contained themes of the medicinal use of cannabis and used data from social media and search engine results. This study analyzed the methodological approaches and limitations of these studies.

**Objective:**

We aimed to examine research approaches and study methodologies that use web-based user-generated text to study the use of cannabis as a medicine.

**Methods:**

We searched MEDLINE, Scopus, Web of Science, and Embase databases for primary studies in the English language from January 1974 to April 2022. Studies were included if they aimed to understand web-based user-generated text related to health conditions where cannabis is used as a medicine or where health was mentioned in general cannabis-related conversations.

**Results:**

We included 42 articles in this review. In these articles, Twitter was used 3 times more than other computer-generated sources, including Reddit, web-based forums, GoFundMe, YouTube, and Google Trends. Analytical methods included sentiment assessment, thematic analysis (manual and automatic), social network analysis, and geographic analysis.

**Conclusions:**

This study is the first to review techniques used by research on consumer-generated text for understanding cannabis as a medicine. It is increasingly evident that consumer-generated data offer opportunities for a greater understanding of individual behavior and population health outcomes. However, research using these data has some limitations that include difficulties in establishing sample representativeness and a lack of methodological best practices. To address these limitations, deidentified annotated data sources should be made publicly available, researchers should determine the origins of posts (organizations, bots, power users, or ordinary individuals), and powerful analytical techniques should be used.

## Introduction

### Medicinal Cannabis Pharmacovigilance

Cannabis has been widely used for a variety of purposes, including medicinal applications, throughout human history. Over the last century, its use has been prohibited in Europe, Northern America, and Australasia [1]. Since 2016, these jurisdictions have incrementally authorized the use of medicinal cannabis for certain conditions [2]. Given the substantial public interest in cannabis as medicine, there is a pressing need to better understand its safety and efficacy.

However, aside from clinical trials, there are scant data regarding the efficacy and side effects of medicinal cannabis [3-6]. One of the main methods for postmarketing safety surveillance of medications is the use of established pharmacovigilance reporting systems, which rely on reporting of adverse events by individuals [7-9]. Cannabis users are often unaware of these systems or the importance of reporting. They may find them too difficult to use or may not want to divulge personal details if these are required [10]. Users may not even think of reporting their side effects because they consider them an inherent experience of cannabis consumption, especially if they are not using an approved medical cannabis product.

Increasing the understanding of the efficacy and safety of cannabis as medicine is warranted because cannabis is a nonstandardized product, given the wide variety in growing conditions and production specifications [11]. This includes variations in climate, soil (or other growth media), water, light, and other factors that affect plant growth. Even if cannabis medicines in a country or state must adhere to mandatory standards (good manufacturing practice), some cannabis users prefer to grow or import their own cannabis [12]. These factors make the systematic assessment of the effectiveness of medical cannabis and its side effects difficult.

### Social Media as a Pharmacovigilance Data Source

To gain additional insights into cannabis use and its effects, researchers are now turning to social media and web-based health forums. These platforms are a place for both patients and the general population to freely express and exchange their experiences and thus provide a valuable additional data source for monitoring public health [13]. Unlike other forms of highly curated data collection methods, such as surveys or interviews, social media provides an organic view of everyday thoughts, behaviors, and activities of people. Therefore, social media has the potential to provide insights beyond the boundaries of targeted investigations, including emergent events, observations of behavioral phenomena and subcultures, and insights for the social sciences [14].

The information contained in social media conversations is voluminous and not only potentially rich in content but also complex and varied. As an unstructured raw data source, credible information may be sparse and difficult to identify; there may be uncertainty about the origin of the data or the population they represent [15]. Furthermore, it is difficult to interpret the informal language and structure of social media posts, which are confounded by many competing sources, such as promotional posts, hashtags, and social media bots [16,17]. Social media bots automatically create content and interact with social media platform users [18]. A study found that between 9% and 15% of Twitter accounts are bots [19]. Notwithstanding these limitations, if these complexities can be successfully navigated, social media has the potential to be a great asset for increased understanding of cannabis as a medicine.

Our previous systematic scoping review [20] used PRISMA (Preferred Reporting Items for Systematic Reviews and Meta-Analyses) guidelines [21] to understand the utility of web-based user-generated text in providing insight into the use of cannabis as a medicine. This paper examines the techniques, analyses, and limitations of these studies.

The objective of this research was to provide a review of studies that have used user-generated data in conjunction with computational methods to understand the medicinal use of cannabis in a population. We addressed the following research questions (RQs):

- RQ1: What consumer-generated data sources are used for studying cannabis?
- RQ2: What common techniques for collection and analysis of data are used?
- RQ3: What are the common limitations and challenges faced by the studies?

## Methods

**Figure 1:**
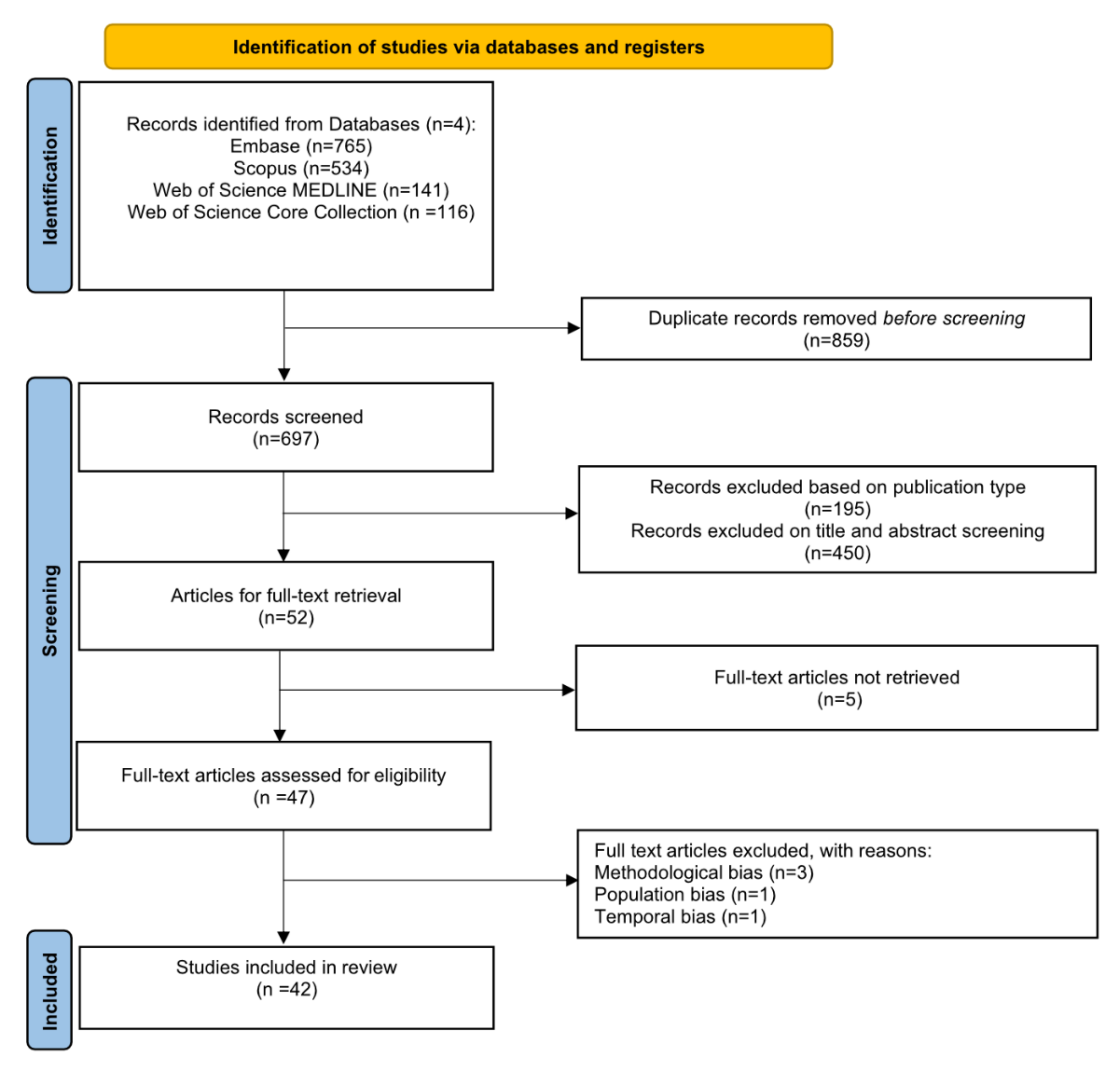
PRISMA (Preferred Reporting Items for Systematic Reviews and Meta-Analyses) flowchart of the study selection process [20].

We searched for English-language studies that were indexed in MEDLINE, Embase, Web of Science, and Scopus databases and published between January 2010 and March 2022. Literature database queries were developed for these 4 databases. See Table S1 in Multimedia Appendix 1 [22-63] for the details of search terms used and Multimedia Appendix 1 Table S2 for the inclusion and exclusion criteria of the selected articles. A summary of the PRISMA flowchart is shown in Figure 1 [20].

## Results

### Overview

Table 1 provides a summary of each article that includes author names, publication year, data source, and duration of data collection, analysis, and number of items analyzed.

The year with the highest number of publications was 2020 (11/42, 26%), followed by 2017 and 2021 (6/40, 14%). Of the 42 studies, 6 (12%) were conducted in 2015 and 2019. The number of publications per year is shown in Table 2.

Regarding data sources, Twitter was used in 40% (17/42) of the reviewed studies, around 3 times the number of studies using either Reddit or web-based forums 14% (6/42). GoFundMe, YouTube, and Google Trends comprised 7% (3/42) of the total. Text was the focus of 83% (35/42) of the studies, whereas the others analyzed trends, videos, search logs, and images. Table 3 shows the distribution of the publications selected per data source.

**Table 1:** Articles included in the review.

Study	Source (duration)	Analysis	Number of items analyzed
McGregor et al [22], 2014	Web-based forums, Facebook, Twitter, and YouTube (not available)	Thematic and content analysis of glaucoma-related posts on the following: Analysis of the nature of the post (personal stories, information sharing or ﬂagging, supportive comments, questions, answers, and general discussions) Sentiment analysis (positive or negative)	3785 items
Cavazos-Rehg et al [23], 2015	Twitter (February to March 2014)	Cannabis-related chatter by influential users on the following: Sentiment analysis by using the Likert scale Thematic analysis of tweets Demographic analysis	7000 tweets
Daniulaityte et al [24], 2015	Twitter (October to December 2014)	US dab-related tweets: Counting and normalizing based on cannabis legalization policy	125,255 tweets (27,018 geolocated tweets)
Gonzalez-Estrada et al [25], 2015	YouTube (June 4-8, 2014)	Content analysis of asthma-related videos on the following: Source: professional society, media, asthma care provider, etc Content: personal experience, medical professional, advertisement, patient education, alternative treatment, or to increase awareness Quality scoring of misleading and useful info Video characteristics or video statistics	200 most viewed videos
Krauss et al [26], 2015	YouTube (January 22, 2015)	Analysis of dabbing-related videos on the following: Characteristics of the people dabbing (age and skills) Characteristics of the session Messages included in the videos	116 videos
Thompson et al [27], 2015	Twitter (March 2012 to July 2013)	Content analysis of cannabis-related tweets and retweets on the following: Adolescence users (age, inferred from the user profile) Sentiment (positive, negative, or unclear) Subject (self, other, general, or subject unclear) Use category (own use, use by others, or not mentioned) Related behaviors (habitual use, social aspect, etc) Positive aspects (better than other drugs and medical use)	36,939 original tweets and 10,000 retweets
Cavazos-Rehg et al [28], 2016	Twitter (January 2015)	Dabbing-related tweets: Thematic analysis of tweets to 7 themes Subanalysis of 1 theme (extreme effects) into physiological or psychological effects Geotagged tweets analysis for number per state Demographic analysis	5000 tweets
Lamy et al [29], 2016	Twitter (May to July 2015)	Content analysis of cannabis edible-related conversations: Tweet sources (media, retail, or users) Sentiment analysis (positive, negative, or neutral) Word frequency analysis Geotagging (policy impact on the volume of tweets)	3000 tweets
Mitchell et al [30], 2016	Web-based forums (October 2014)	Thematic analysis of ADHD^a^ and cannabis web-based forum posts on the following: Impact of cannabis on ADHD symptoms (therapeutic, harmful, both, and none) Other domains (mood, psychiatric conditions, and other [sleep]) Comments about cannabis as medicinal (more effective than other ADHD medications, less effective, or not legal)	268 threads
Andersson et al [31], 2017	Web-based forums (April 18-19, 2016)	Thematic analysis of conversations on headache-related posts	32 topics
Dai and Hao [32], 2017	Twitter (August 2015 to April 2016)	Naive Bayes classifier on PTSD^b^ and cannabis-related tweets: Sentiment analysis Analysis of prevalence of support of cannabis use for PTSD in association with state level legislation and socioeconomic factors	66,000 cannabis-related and 31,184 geolocated tweets
Greiner et al [33], 2017	Web-based forums (November 2014 to March 2015)	Content analysis of cannabis help forums on the following: Fields of interest (illness-related, social, ﬁnancial, and legal issues) Self-help mechanisms (exchange of information, emotional support, group support) Analysis of sex and age when available Highly involved vs moderately involved users	717 posts
Turner and Kantardzic [34], 2017	Twitter (August 2015 to April 2016)	Supervised and unsupervised machine learning techniques of cannabis-related tweets: Binary classification to identify marijuana-related tweet Topic modeling User social network analysis Spatiotemporal analysis of conversations	40,509 geolocated tweets
Westmaas et al [35], 2017	Web-based forums (January 2000 to December 2013)	Topic modeling of Cancer Survivors Network: Analyze smoking or cessation-related content Analysis to determine the overall context in which these discussions occurred	468,000 posts
Yom Tov and Lev Ran [36], 2017	Bing logs (November 2016 to April 2017)	Statistical analysis of cannabis-related query logs	Not available
Cavazos-Rehg et al [37], 2018	YouTube (June 10-11, 2015)	Cannabis review web-based videos: Sentiment analysis Physical or mental effects; is it promotional, encourage follow-up; depiction of consumption; video details and engagement statistics Current users survey (demographics, reason for use, and use of reviews)	83 videos
Glowacki et al [38], 2018	Twitter (August to October 2016)	Statistical analysis on opioid-related tweets: Clustering algorithm to find topics Analysis of trending hashtags, top inﬂuencers, and location of tweets	73,235 tweets
Meacham et al [39], 2018	Reddit (January 2010 to December 2016)	Analysis of modes of cannabis use mentions on Twitter on the following: Most frequent words Mentions of adverse effects Subjective highness	400,000 posts
Leas et al [40], 2019	Google Trends (January 2004 to April 2019)	Analysis on CBD^c^ and cannabidiol terms to evaluate public interest	Not available
Meacham et al [41], 2019	Reddit (January 2017 to December 2019)	Content analysis of dabbing-related questions on the following: Topics of questions After engagement and the types and sentiment of information	193 questions
Nasralah et al [42], 2019	Twitter (January 2015 to February 2019)	Analysis of opioid-dependent user’s tweets: Thematic analysis of conversations Demographic analysis	20,609 tweets
Pérez-Pérez et al [43], 2019	Twitter (February to August 2018)	Lexicon- and rule-based analysis of bowel disease tweets on sentiments, network, gender, geolocation, symptoms, and food	24,634 tweets
Shi et al [44], 2019	Google Trends and Buzzsumo (January 2011 to July 2018)	Google Trends analysis on cancer therapies to evaluate interest in cannabis vs other therapies	Not available
Allem et al [45], 2020	Twitter (May to December 2018)	Topic analysis of cannabis-related tweets	60,861 nonbot and 8874 bot tweets
Janmohamed et al [46], 2020	Blogs, news, forums, and <1% other (August 2019 to April 2021)	Topic modeling on vaping-related conversations: Analysis of word prevalence Analysis of change of topics over time	4,027,172 documents or blogs
Jia et al [47], 2020	Google, Facebook, and YouTube (September 2019)	Content analysis of glaucoma and CBD posts on the following: General discussion, information sharing, personal story, question, answer, and moderator comment Quality of information Source of information being professional or not and whether an opinion on glaucoma and medical cannabis use was expressed Analysis of professional accounts	51 Google websites, 126 Facebook posts, and 37 YouTube videos
Leas et al [48], 2020	Reddit (January 2014 to August 2019)	Content analysis of reasons for CBD use: Reasons for personal use (condition and wellness) Analysis based on categorized diagnosable conditions	104,917 posts
Merten et al [49], 2020	Pinterest (July 31, August 18, and September 1, 2018)	Content analysis of CBD and cannabidiol posts on the following: Mentions of mental and physical benefits Emotional appeal analysis Engagement statistics	1280 pins
Mullins et al [50], 2020	Twitter (June to July 2017)	Analysis of Ireland pain-related tweets on: Topic analysis: sentiment analysis, analysis of most frequently occurring keywords, demographic analysis, and personal use analysis	941 tweets
Saposnik and Huber [51], 2020	Google Trends (January 2004 to December 2019)	Google Trends analysis on autism and cannabis to analyze trends in search volume about the causes and treatments of Autism spectrum disorder over time	Not available
Song et al [52], 2020	GoFundMe (January 2012 to December 2019)	Content analysis of alternative medicine and cancer campaigns on the following: Themes of patient narratives Types of alternative treatments used Demographics (gender, cancer type, cancer stage, insurance status, past treatment, future treatment, and alternative treatment)	1474 campaigns
Tran and Kavuluru [53], 2020	Reddit and or FDA comments (January to April 2019)	Content analysis on CBD posts for therapeutic effects and popular modes of consumption compared with FDA^d^ comments	64,099 Reddit and 3832 FDA comments
van Draanen et al [54], 2020	Twitter (January 2017 to June 2019)	Cannabis-related US and Canada posts: Topic modeling Sentiment analysis based on cannabis legalization policies	1,200,127 tweets
Zenone et al [55], 2020	GoFundMe (January 2017 to March 2019)	Thematic analysis of cancer and cannabis campaigns: Efficacy claims Treatment regimen classiﬁcation CBD efﬁcacy presentation Content analysis for Other: cancer stage, raised money, and number of donors	155 campaigns
Pang et al [56], 2021	Twitter (December 2019 to December 2020)	Thematic analysis of pregnancy- and cannabis-related tweets for safety during pregnancy, safety postpartum, and pregnancy-related symptoms	17,238 tweets
Rhidenour et al [57], 2021	Reddit (January 2008 to December 2018)	Thematic analysis of veteran’s cannabis posts on the following: Point of view, reasons for use, prescription drug use, or other substance use Test, legality, legal policy, and doctor-patient conversation	974 posts
Smolev et al [58], 2021	Facebook (November 2018 to November 2019)	Thematic analysis of traumatic brachial plexus injury posts on: antiopioid sentiment, preference for alternative options, and antigabapentin sentiment	7694 posts
Soleymanpour et al [59], 2021	Twitter (July 2019)	Analysis of CBD marketing tweets and therapeutic claims	2,200,000 tweets
Zenone et al [60], 2021	GoFundMe (June 2017 to May 2019)	Thematic analysis for informational pathways: self-directed research, recommendations from a trusted care provider, and insights shared by someone associated with or influencing the crowd funders personal network Content analysis for intended outcome, social media shares, number of donors, total requested, and total received	164 campaigns
Turner et al [62] 2021	Twitter (October 2019 to January 2020)	Analysis of personal and commercial CBD-related tweets; term and sentiment analysis	167,755 personal 143,322 commercial tweets
Allem et al [61], 2022	Twitter (January to September 2020)	Analysis of cannabis-related conversation for health-related motivations or perceived adverse health effects	353,353 tweets
Meacham et al [63] 2022	Reddit (December 2015 to August 2019)	Analysis of cannabis-related posts from an opioid use and an opioid recovery subreddit	908 posts from opioid recovery subreddits and 4224 posts from opioid use subreddits

^a^ADHD: attention-deficit hyperactivity disorder.

^b^PTSD: posttraumatic stress disorder.

^c^CBD: cannabidiol.

^d^FDA: Food and Drug Administration.

**Table 2:** Publications per year (n=42).

Year	Count, n (%)
2014	1 (2)
2015	5 (12)
2016	3 (7)
2017	6 (14)
2018	3 (7)
2019	5 (12)
2020	11 (26)
2021	6 (14)
2022	2 (5)

**Table 3:** Publications per data source (n=42).

Source	Count, n (%)
Twitter	17 (41)
Reddit	6 (14)
Web-based forums	6 (14)
GoFundMe	3 (7)
YouTube	3 (7)
Google Trends	3 (7)
Google, Facebook, and YouTube	1 (2)
Bing Search Engine	1 (2)
Facebook	1 (2)
Pinterest	1 (2)

### Social Media Data Collection Strategies

Some studies obtained all their associated data from a specific subreddit [48,53,57] or a web-based forum [35] and subsequently sampled the data. Of 42 studies, 1 (2%) Twitter study collected tweets using a geolocation boundary box and then filtered the data for cannabis-related keywords [54].

Keyword-based filtering was used by many studies. Terms used for filtering were either common expressions for cannabis from dictionaries, such as Urban Dictionary, or were based on similar research in this domain. Of the 42 studies,1 (2%) study [36] used Urban Dictionary and web forums to create a comprehensive list of 123 terms related to cannabis consumption. Another study [57] first found all the terms related to marijuana by searching on Thesaurus.com and then used the word embedding likeness perusal software [64] to generate synonyms.

In a nonmedical cannabis-related study, word embeddings created from Twitter and Reddit data sets discovered synonyms and slang terms that could not be identified using other means. The study recommends this method of synonym discovery in advance for any data collection based on keyword filtering [65].

Of the 42 studies, 3 (7%) studies were user focused, with data derived from specific highly influential users [23], opioid-dependent users [42], or a US veteran-specific subreddit [57].

The largest data set manually annotated by the researchers was collected using cannabis-related keywords and consisted of 36,939 original tweets and 10,000 retweets [27]. Apart from that study, the average size of annotated data sets was approximately 1450 records. Of the 42 studies, 2 (5%) studies [23,28] used crowdsourcing services to annotate tweets, whereas the rest conducted in-house annotation. The duration of data collection ranged from 1 month to 6 years. Of the 42 studies, 2 (5%) of these studies made their annotated data available to other researchers [30,60].

### Types of Analysis

#### Overview

The studies included in this review used a variety of analytical methods, including qualitative analysis, quantitative content analysis, machine learning, rule-based, and statistical analysis. The types of analysis include sentiment assessment, thematic analysis, content analysis, named entity recognition, social networks, and geographic analysis. Table S3 in Multimedia Appendix 1 summarizes the analyses.

#### Discovering Themes

Themes were identified in 62% (26/42) of the studies. Manual coding of the themes was performed by 69% (18/26) of the studies, either by using pre-existing categories or by observing a sample of the data and generating a codebook [22,23,25,26,28,30,31,37,41,47-49,52,55-58,60]. Of the 26 studies, 2 (7%) studies used the services of social media data analytics companies [42,50].

Of the 26 studies, 4 (15%) studies used topic modeling to infer themes or topics [34,35,46,54]. The algorithm of choice for this task is the latent Dirichlet allocation [66]. The choice of the number of topics was based on intrinsic evaluation metrics (eg, coherence and perplexity) and iterative qualitative analysis informed by prior experience with topic models. Of the 26 studies, 1 (4%) study used temporal topic modeling techniques to study changes in topics over time, with the goal of analyzing how web-based vaping narratives changed during the COVID-19 pandemic [46].

Of the 26 studies, 1 (4%) study identified themes by using rule-based methods. Frequency counts of the most common unigrams and bigrams were generated and formed the basis of the topics [45]. Another study used SAS Text Miner software, a text-topic node algorithm, to discover topics [38].

#### Demographic Analysis

Socioeconomic and demographic analyses of the study population were performed in 26% (11/42) of the studies. Of the 11 studies, 2 (27%) studies used the provided gender, age, and other user characteristics from user profiles or inferred from posts by users [33,52]. Of the 11 studies, 2 (27%) video-based studies used the perceived age and gender of the subjects after observing the videos [25,26].

Of the 11 studies, 2 (18%) studies that used social media analytics providers obtained age and gender data by using the supplied analysis [42,50]. Of the 11 studies, 2 (18%) of the Twitter-based studies used a commercial tool called DemographicsPro, which uses proprietary algorithms to infer user demographic characteristics [23,28]. Other studies used existing census data [32], demographic information obtained from survey data [37], and a 2-step method based on a gender-name lexicon and a face recognition algorithm applied to users’ profile information to identify the users’ gender [43].

#### Geographic Analysis

Geolocation data analysis was performed in 40% (17/42) of the studies. User profiles or message metadata were used in 52% (9/17) of the studies [24,29,32,34,36,43,54,55,60]. Of the 17 studies, 2 (12%) studies used information provided by social media analytics companies [38,50]. The DemographicsPro tool was used in 5% (1/17) of studies [28]. Of the 17 studies, 3 (17%) studies used location information provided by Google Trends [40,44,51]. Another (1/17, 5%) study collected geographical information from survey data [37]. Of the 17 studies,1 (5%) video-based study used the geographic location of video channels [26].

#### Sentiment Assessment

An individual’s perception of a topic can be characterized as having a positive, negative, or neutral sentiment. The analysis of these sentiments is often performed using automated language tools and is named “sentiment analysis” [67].

Out of the 12 studies that performed sentiment analysis, 5 (42%) used automated methods. Of the 12 studies, 1 (8%) study trained a binary Naive Bayes classifier on a sample of 1000 “marijuana” related tweets to classify posts into 2 opinion polarities, positive and negative or neutral [32]. Another study used sentiment analysis provided by a social media analytics company [50]. Of the 12 studies, 3 (25%) studies used Valence Aware Dictionary and Sentiment Reasoner (VADER) [68], a lexicon and rule-based sentiment analysis tool [43,54,62]. The VADER performance was compared with in-house machine learning classifiers trained on 3000 manually coded cannabis-related tweets, which showed a 30% performance improvement over VADER. Although VADER is widely used for general tweet sentiment analysis, its performance suffers in substance-use-related domains where negative words are often used to carry positive sentiments. For example, “I took CBD oil, that stuff was bad” [69]—in this sentence, “bad” actually means good.

#### User Analysis

For conducting user analysis, 57% (24/42) of the studies examined either the subject of the posts, as from individuals or others (ie, from self, retail, media, or professionals), or who the post was about (self, others, or general) [22,23,25-29,33,37,41-43,45,47-50,52,55,57,58,60-62].

When manual data labeling was performed, the determination of both the poster and subject of the post was part of the labeling process. Self-reporting and self-use were easily determined by observation of videos, as were most texts based on the structure of the language. For example, a study [27] first identified whether the subject of the tweet was about the self, other, or general and then identified whether the tweets were about actual cannabis use. This study included further categories of tone, related behavior, perceived impact, and social context. Automated labeling approaches look for phrases that indicate self-reporting. For example, a study on opioid addiction [42] looked for phrases such as “I am addicted” and “I have been addicted” in the context of opioid mentions. Classifiers were used in another study [59] to separate marketing tweets from nonmarketing tweets; however, their focus was on marketing tweets.

None of the studies used advanced natural language processing techniques to establish subjects and personal mentions. Social media bots are automated accounts that generate artificial activities on social media platforms [18]. Bot detection was used in only 4% (1/24) of studies, which used Twitter as a data source [45].

#### Other Analyses

Of the 42 studies, 2 (5%) studies examined the social networks of contributors to conversations. This allowed the identification of target communities and user interactions [34,43]. Of the 42 studies, 3 (7%) studies examined the impact of governmental cannabis legalization policies on the sentiments and opinions of people or on the volume of social media posts [24,28,54]. Term frequency and count analysis of words and phrases was performed in 12% (5/42) of studies [29,39,50,62,63].

#### Ethical Considerations

Institutional review boards (or their equivalents) ensured that research using human participants is conducted in an ethical manner [70]. Approval for and overseeing of a study by an institutional review board ensures that researchers adopt an ethically appropriate research protocol that respects the rights and interests of social media users; 62% (26/42) of the studies mentioned an ethics approval review being sought or the study being exempt from ethics requirements. There was no mention of ethics approval in 38% (16/42) of the studies.

#### External Validity

The use of standard reporting systems, such as the US Food and Drug Administration reports, helps to assess whether social media research findings can be generalized to real-world data. When a suitable ground-truth data set is not available, validating results against >1 social media platform improves the generalizability and validity of the results. Only a few studies used >1 social media data source or validated their findings against other data sources. Of the 42 studies, 2 (5%) studies used Food and Drug Administration data as an external ground-truth data source to validate their results [36,53]. Of the 42 studies, 1 (2%) study analyzed several web-based forums [31], and 2 (5%) other studies used several social media platforms as their data sources [22,47].

## Discussion

In this study, we reviewed the technical aspects of peer-reviewed published works that used social media and other forms of user-generated data to understand the medicinal use of cannabis. All the studies concluded that these consumer-generated data sources are useful and provide a complementary resource for studying cannabis and medical conditions for which cannabis is used.

### Principal Findings

The findings of this study are presented by answering the RQs.

#### RQ1: What Consumer-Generated Data Sources Are Used for Studying Cannabis?

Sources of consumer-generated data for cannabis research used by the reviewed studies include social media platforms, such as Twitter, Reddit, and YouTube; search queries, including Google Trend and Bing query logs; and web-based forums, crowdfunding platforms, blogs, and websites. Twitter was used in most of the studies. One of the studies concluded that, compared with unmoderated platforms, moderated sites focused more on evidence-based information and controlled misleading content [22].

#### RQ2: What Common Techniques for Collection and Analysis of Data Are Used?

Some studies have used social media analytics companies for some or all of their data collection and processing tasks. Other studies used application program interfaces to interact with Twitter and Reddit. Although Facebook allows researchers to access public posts from public pages through a dedicated platform [71], 2% (1/42) of studies [58] analyzed private Facebook posts—the method used to obtain data was not reported.

Approximately half of the studies used data sets of <8000 records and many of them used 1000 records. These studies either focused on understanding the characteristics and needs of users or the quality of information on the web, or they were directed by an RQ such as “Are individuals using CBD for diagnoseable conditions which have evidence-based therapies?” These analyses play a critical role in understanding the domain but are difficult to replicate and generalize.

More recent neural network–based natural language processing techniques have not been used in the studies in this review. These modern machine learning methods have the advantage that they require minimal data preparation and are characterized by the capacity to learn the nuance of language. However, to function effectively, they typically require high-quality annotated data—a scarce and expensive resource. Textual social media data are highly amenable to these techniques. Creating and sharing deidentified annotated data sets for this purpose should be encouraged within appropriate ethical, regulatory, and legal frameworks [72].

#### RQ3: What Are the Common Limitations and Challenges Faced by the Studies?

These limitations are mentioned in order of frequency.

##### Sample Representativeness

Most research on social media uses samples of available data. However, the extent to which the data samples are representative of the general population is often unclear. The limiting factors mentioned in these studies include sampling bias that is introduced as a result of the choice of keywords, data collection duration, and population biases.

Population biases often refer to the demographic composition of people using social media platforms being different from the general population and the difficulties in determining the demographic characteristics of users. Accessing accurate geographical locations has also been mentioned in previous studies. Obtaining these data is limited because even when users explicitly include demographic information (eg, with Facebook) or geographical information in their posts or profiles, these may be fabricated.

The choice of platform itself also imposes limitations. For instance, platform-specific features, such as sampling strategies, limit the amount of data that can be collected and the behavior and conversation of users depending on the platform or context. Of the 42 studies,1 (2%) study mentioned that the forums they investigated could be very procannabis and are likely inhabited by more experienced cannabis users [41]. Another study stated that individuals posting on YouTube about cannabis are likely to seek social networking opportunities [37].

Complications also arise because platform-specific algorithms spot and further promote popular themes and users to deliberately manage behavior and attract more platform engagement. This needs to be ameliorated by detecting and accounting for the algorithms and potentially by sampling from >1 platform.

##### Methodology Constraints

The use of small data sets by some of the studies impacts the generalizability of the results, and some of the researchers acknowledged this and indicated a plan to replicate their studies with more data and the use of automated methods. Consequently, we observed that although such studies may be sampling social media data for hypothesis generation, they do not leverage one of the most important features of social media data, which is the ability to observe the continuous generation of big data to create long-term data-centric insights [73].

Biases that could have been introduced by the choice of theme were also mentioned in the studies. Most researchers have attempted to mitigate this by creating annotation guidelines, having >1 person labeling data, and resolving disagreements.

##### Actual Use Detection

A limitation mentioned by several studies is that web-based search activities and social media posts containing cannabis-related keywords do not necessarily represent the actual use of cannabis by the poster. Depending on the context and goals of the research (for instance, if the research seeks to study a cannabis-consuming population), advanced text processing techniques are required to establish when personal cannabis use can be inferred. For such studies, establishing its use should be a crucial initial step. However, the detection of personal use is challenging, especially in the informal, diverse, and specialized language used by niche communities.

##### Source Identification

Identifying the source of posts (ie, whether they were generated organically by individual users or by organizations or bots) was a commonly mentioned limitation. Content generated by health and commercial organizations, power users, and nonindividual accounts was understood to comprise a considerable amount of social media post volume on the web.

### Limitations

This review used 4 literature databases in the search process to allow the maximum coverage of existing publications. However, we cannot be certain that we have covered all relevant publications. The choice of keywords for the literature search could also have impacted capturing all the relevant studies in this domain, for instance, *infodemiology* and *infoveillance* were not in the keywords. Articles included in this study were selected following a systematic approach and underwent a bias assessment for quality; however, biases could not be completely avoided. This study was also limited to English-language articles.

### Conclusions

The number of studies in this field has steadily increased over the last few years. Social media conversations are wide ranging and offer opportunities for insights that cannot be obtained through formal information gathering. Researchers have realized the value of social media conversations as a place for users to freely express their experiences and concerns without risking judgment or penalty and that social media is the natural forum for many users of cannabis as medicine to share their insights into the benefits and issues they experience and perceive.

Manual qualitative analysis, statistical analysis, supervised and unsupervised machine learning, and rule-based methods are among methodologies used in these studies. Analyses of social media data that are limited to small data samples, although providing an effective means of hypothesis generation, are difficult to reliably reproduce and generalize. Where possible, the sharing of high-quality deidentified annotated data to allow the use of generalizable analytical techniques should be encouraged to advance this field.

To improve their validity and generalizability, studies could add additional social media data sources and check their results against established reporting systems. Studies could take advantage of emerging data analysis strategies that leverage big data, such as deep learning and transfer-learning-based approaches.

## Appendix

Multimedia Appendix 1 Supporting information (review keywords, inclusion and exclusion criteria, papers summary).

## Abbreviations

PRISMA: Preferred Reporting Items for Systematic Reviews and Meta-Analyses
RQ: research question
VADER: Valence Aware Dictionary and Sentiment Reasoner
