# Supplementary material for: Consumer-Generated Discourse on Cannabis as a Medicine: Scoping Review of Techniques
Source: J Med Internet Res. 2022 Nov 16;24(11):e35974. doi: 10.2196/35974 (PMC9713623; doi:10.2196/35974)
Supplement: Multimedia Appendix 1 [file jmir_v24i11e35974_fig.ocx]

# Multimedia Appendix 1

Supporting information (review keywords, inclusion and exclusion criteria, papers summary).

Table S1— Categories of literature database query terms used for finding relevant papers to be used in the review. The first three categories targeted papers that used social media as a data source and that included the use of cannabis and health related effects. The fourth category used search engine queries as a data source where cannabis was mentioned.

| **Category 1 - Social media, cannabis, and medical terms** | | |
| --- | --- | --- |
| *Social media Keywords* | *Cannabis keywords* | *Medical keywords* |
| ‘Social media’ OR twitter OR reddit OR instagram OR youtube OR pinterest OR facebook OR ‘social network forum’ OR ‘Online health community’ OR ‘message board’ | cannabis OR marijuana OR cannabinoids OR delta-9-tetrahydrocannabinol OR cannabidiol OR cbd OR cbg OR cbn OR thc OR weed | medical OR medicinal OR patient OR patients OR medicine OR doctor OR position OR care OR therapy OR therapeutic |
| **Category 2 - Social media, cannabis and Psychiatric disorders** | | |
| *Social media Keywords* | *Cannabis keywords* | *Psychiatric disorders* |
| ‘Social media’ OR twitter OR reddit OR instagram OR youtube OR pinterest OR facebook OR ‘social network forum’ OR ‘Online health community’ OR ‘message board’ | cannabis OR marijuana OR cannabinoids OR delta-9-tetrahydrocannabinol OR cannabidiol OR cbd OR cbg OR cbn OR thc OR weed | depression OR depressive OR ‘mental illness*’ OR ‘mental disorder*’ OR ‘mental health’ OR ‘mood disorder*’ OR ‘affective disorder*’ OR anxi* OR ‘panic disorder’ OR ‘obsessive compulsive’ OR adhd OR ‘attention deficit’ OR phobi* OR bipolar OR psychiat* OR psychological OR psychosis OR psychotic OR schizophr* OR ‘severe mental*’ OR ‘serious mental*’ OR antidepress* OR antipsychotic* OR ‘post traumatic*’ OR ‘personality disorder*’ OR stress |
| **Category 3 - Social media, cannabis and various medical (non-psychiatric) conditions illnesses** | | |
| *Social media Keywords* | *Cannabis keywords* | *Medical conditions* |
| ‘Social media’ OR twitter OR reddit OR instagram OR youtube OR pinterest OR facebook OR ‘social network forum’ OR ‘Online health community’ OR ‘message board’ | cannabis OR marijuana OR cannabinoids OR delta-9-tetrahydrocannabinol OR cannabidiol OR cbd OR cbg OR cbn OR thc OR weed | Pain, Opioid, Alzheimer, sleep OR insomnia, inflammatory, arthritis, Multiple Sclerosis, Endometriosis |
| **Category 4 - Search engine queries and cannabis keywords** | | |
| *Search Engine keywords* | *Cannabis keywords* | |
| ‘Search engine’ OR ‘search log’ OR ‘search queries’ OR ‘online search’ OR ‘internet Search’ OR ‘web search’ | cannabis OR Marijuana OR Cannabinoids OR Delta-9-Tetrahydrocannabinol OR Cannabidiol OR CBD OR CBG OR CBN OR thc OR weed | |

Table S2 — Inclusion and exclusion criterion

| **Criteria** | **Included** | **Excluded** |
| --- | --- | --- |
| Study type | Primary research studies. | Editorials, letters, commentaries, book chapters, and studies where the full text of the publication was not available |
| Intervention | Online user-generated text as a data source. | Studies that used social media for recruiting participants.  Studies that used bots or autonomous systems as the main data source |
| Outcome | Research that was either directly focused on cannabis and cannabis products that have an impact on health or were health-related studies that found medicinal use of cannabis. | Studies primarily focused on electronic nicotine delivery systems adapted to deliver cannabinoids.  Studies that focused exclusively on synthetic cannabis. |
| Language | Published in English |  |
| Timeframe | Jan 1975-March 2022 |  |

Table S3 — Analysis performed in each study

| **Study** | **Themes Analysis** | **Demographics Analysis** | **Geographics Analysis** | **Sentiment Assessment** | **User Analysis** | **Ethics** | **External validity** | **Social Network** | **Term Analysis** |
| --- | --- | --- | --- | --- | --- | --- | --- | --- | --- |
| McGregor et al., 2014 [22] | Y |  |  |  | Y | Y | Y |  |  |
| Cavazos-Rehg et al., 2015 [23] | Y | Y |  | Y | Y | Y |  |  |  |
| Daniulaityte et al., 2015[24] |  |  | Y |  |  | Y |  |  |  |
| Gonzalez-Estrada et al., 2015 [25] | Y |  |  |  | Y | Y |  |  |  |
| Krauss et al., 2015[26] | Y | Y | Y |  | Y |  |  |  |  |
| Thompson et al., 2015 [27] |  | Y |  | Y | Y | Y |  |  |  |
| Cavazos-Rehg et al., 2016 [28] | Y | Y | Y |  | Y |  |  |  |  |
| Lamy et al., 2016 [29] |  |  | Y | Y | Y | Y |  |  | Y |
| Mitchell et al., 2016 [30] | Y |  |  | Y |  | Y |  |  |  |
| Andersson et al., 2017 [31] | Y |  |  |  |  | Y |  |  |  |
| Dai & Hao, 2017 [32] |  | Y | Y | Y |  |  |  |  |  |
| Greiner et al., 2017 [33] |  | Y |  |  | Y | Y |  |  |  |
| Turner, 2017 [34] | Y |  | Y |  |  |  |  | Y |  |
| Westmaas et al., 2017 [35] | Y |  |  |  |  | Y |  |  |  |
| Yom Tov & Lev Ran, 2017 [36] |  |  | Y |  |  |  | Y |  |  |
| Cavazos-Rehg et al., 2018 [37] | Y | Y | Y | Y | Y | Y |  |  |  |
| Glowacki et al., 2018 [38] | Y |  | Y |  |  |  |  |  |  |
| Meacham et al., 2018 [39] |  |  |  |  |  | Y |  |  | Y |
| Leas et al., 2019 [40] |  |  | Y |  |  | Y |  |  |  |
| Meacham et al., 2019 [41] | Y |  |  | Y | Y | Y |  |  |  |
| Nasralah et al., 2019 [42] | Y | Y |  |  | Y |  |  |  |  |
| Pérez-Pérez et al., 2019 [43] |  | Y | Y | Y | Y |  |  | Y |  |
| Shi et al., 2019 [44] |  |  | Y |  |  |  |  |  |  |
| Allem et al., 2020 [45] | Y |  |  |  | Y | Y |  |  |  |
| Janmohamed et al., 2020 [46] | Y |  |  |  |  | Y |  |  |  |
| Jia et al., 2020 [47] | Y |  |  |  | Y | Y | Y |  |  |
| Leas et al., 2020 [48] | Y |  |  |  | Y | Y |  |  |  |
| Merten et al., 2020 [49] | Y |  |  | Y | Y |  |  |  |  |
| Mullins et al., 2020 [50] | Y | Y | Y | Y | Y |  |  |  | Y |
| Saposnik & Huber, 2020 [51] |  |  | Y |  |  |  |  |  |  |
| Song et al., 2020 [52] | Y | Y |  |  | Y |  |  |  |  |
| Tran & Kavuluru, 2020 [53] |  |  |  |  |  | Y | Y |  |  |
| Van Draanen et al., 2020 [54] | Y |  | Y | Y |  | Y |  |  |  |
| Zenone et al., 2020 [55] | Y |  | Y |  | Y | Y |  |  |  |
| Pang et al., 2021 [56] | Y |  |  |  |  | Y |  |  |  |
| Rhidenour et al., 2021 [57] | Y |  |  |  | Y |  |  |  |  |
| Smolev et al., 2021 [58] | Y |  |  |  | Y |  |  |  |  |
| Soleymanpour al., 2021 [59] |  |  |  |  |  |  |  |  |  |
| Zenone et al., 2021 [60] | Y |  | Y |  | Y | Y |  |  |  |
| Allem et al., 2022 [62] |  |  |  |  | Y | Y |  |  |  |
| Turner et al. 2021 [61] |  |  |  | Y |  | Y |  |  | Y |
| Meacham et al. 2022 [63] | Y |  |  |  |  | Y |  |  | Y |
